# MTMixG-Net: mixture of Transformer and Mamba network with a dual-path gating mechanism for plant gene expression prediction

**DOI:** 10.3389/fpls.2025.1718258

**Published:** 2025-11-19

**Authors:** Fei Guo, Wenjuan Li, Aihong Lu, Rongzhen Feng, Wu Fang

**Affiliations:** School of Information Technology, Suzhou Institute of Trade and Commerce, Suzhou, China

**Keywords:** plant gene expression, transcriptional regulation, Transformer, Mamba, gate mechanism

## Abstract

Accurate prediction of plant gene expression is essential for elucidating the regulatory mechanisms underlying plant development and stress adaptation. Traditional experimental approaches such as microarrays and RNA sequencing have provided valuable insights but remain limited in capturing the complexity and diversity of genomic regulation. Recent advances in deep learning have shown promise, yet existing models often struggle to generalize across species and to efficiently model long-range dependencies within genomic sequences. To address these challenges, we propose MTMixG-Net, a novel deep learning framework that integrates Transformer and Mamba architectures with a gating mechanism for enhanced gene expression prediction. MTMixG-Net consists of three main modules: the mixture of Transformer and Mamba encoder (MTMixEnc), the dual-path gating mechanism (DPGM), and the residual CNN chain (ResCNNChn). The MTMixEnc combines the self-attention capacity of Transformers with the state-space efficiency of Mamba to capture multi-scale regulatory dependencies while maintaining low computational complexity. The DPGM adaptively refines feature selection through dynamic gating, allowing the model to focus on the most informative representations. Finally, the ResCNNChn leverages a sequence of residual CNN blocks to extract high-level features and further boost predictive accuracy. We validate MTMixG-Net on multiple plant genomic datasets, demonstrating its superior accuracy and computational efficiency compared to existing methods. Our results highlight the potential of MTMixG-Net as a powerful tool for advancing plant genomics research and crop improvement strategies.

## 1 Introduction

Accurate identification and functional characterization of cis-regulatory elements (CREs) are essential for advancing genetic improvement in plants. By interacting with transcription factors (TFs), CREs modulate gene expression and thereby influence critical agronomic traits, including growth, stress tolerance, and yield (Adamo et al., 2021; Rodríguez-Leal et al., 2017). Understanding CRE functions facilitates the development of precise molecular markers and informs targeted gene-editing strategies, ultimately accelerating crop improvement. Nevertheless, the intricate architecture of plant genomes, especially the largely uncharacterized roles of non-coding regions, presents major challenges for conventional approaches to regulatory element discovery and annotation.

Traditionally, gene expression prediction has relied on experimental techniques such as microarrays and RNA sequencing (Bai et al., 2024; Kim et al., 2024; Deshpande et al., 2023; Li et al., 2022b). However, these approaches are often expensive, time-consuming, and limited in their ability to capture the full complexity of gene regulation. Recent advances in machine learning, particularly deep learning, offer powerful alternatives to overcome these limitations (Zumkeller et al., 2025; Yan et al., 2025a; Jin et al., 2025a; Zhou et al., 2025; Jin et al., 2025b). By leveraging large-scale datasets, deep learning models can uncover hidden regulatory patterns, thereby improving prediction accuracy and enabling scalable analysis. For example, sequence-to-expression models such as Basenji (Kelley et al., 2018) and its successors have learned regulatory grammars directly from long DNA sequences, allowing the prediction of cell-type-specific transcriptional profiles from sequence data alone. These models demonstrated that convolutional architectures can effectively capture distal enhancer signals at scales of up to 100 kb. Similarly, explainable convolutional neural networks (CNNs) have decoded cis-regulatory elements involved in tomato fruit development, enabling the prediction of tissue- and stage-specific expression and highlighting the applicability of sequence-based models to crop genomes (Herrera-Ubaldo, 2022). Barbadilla-Martínez et al. (2025) further demonstrated that deep learning can accurately predict gene expression directly from DNA sequences, modeling regulatory grammars and revealing the functional impact of non-coding variants. DeepMethyGene (Yan et al., 2025b) achieved superior performance in expression prediction by incorporating methylation features, especially in genomic contexts where methylation sites are sparse or near transcription start sites (TSS). DeepCBA (Wang et al., 2024) combined CNNs, BiLSTMs, and self-attention mechanisms to integrate DNA sequence and chromatin interaction data, achieving state-of-the-art performance in maize expression prediction. Similarly, the DeepTGI framework (Liu et al., 2024) utilized autoencoders with self-attention to infer transcription factor-gene interactions, enhancing regulatory interpretability.

Despite these advances, modeling long-range regulatory interactions remains a significant challenge. To address this, Honig et al. (2024) proposed the genetic sequence token alignment (GTA) approach, which aligns genomic features with natural language tokens. This alignment enables symbolic reasoning via pretrained language models, thereby improving the interpretability of long-range dependencies. However, despite progress in deep learning-based gene expression prediction and transcriptional regulation analysis (Eapen, 2025; Su et al., 2025), several challenges persist that limit the full potential of these models in plant genomics. First, the interactions between genes and their regulatory elements are highly complex and context-dependent. Traditional models often fail to capture the nonlinear and dynamic nature of these relationships, leading to inaccurate predictions (Batbaatar and Ryu, 2025). The key difficulty lies in designing models that can accurately represent these intricate dependencies while considering environmental and external factors. Current deep learning techniques still face significant challenges in modeling these relationships in a biologically meaningful way (Li et al., 2022a). Second, CNNs are primarily designed to detect local patterns through convolutional filters (Muller et al., 2019). While effective at identifying motifs and sequence fragments, they struggle to capture long-range dependencies between distal genes and regulatory elements, which are crucial for accurate expression prediction. Third, Transformer architectures (Vaswani et al., 2017; Consens et al., 2023; Kwak et al., 2024) have shown strong capabilities in modeling long-range dependencies within a single sequence. However, their quadratic complexity and uniform attention mechanism hinder their ability to efficiently capture multi-scale hierarchical relationships inherent in genomic data (Gong et al., 2025). Gene regulation spans multiple biological layers, including promoter-TF interactions, chromatin architecture, and epigenetic modifications, many of which are not adequately addressed by standard Transformer models.

To address these challenges, we propose MTMixG-Net, a novel mixture of Transformer and Mamba networks with a dual-path gating mechanism, specifically designed for plant gene expression prediction. MTMixG-Net consists of three core modules: the mixture of Transformer and Mamba encoder (MTMixEnc), the dual-path gating mechanism (DPGM), and the Residual CNN Chain (ResCNNChn). The MTMixEnc module integrates the self-attention mechanism of Transformers with the hierarchical state-space structure of Mamba, enabling the model to capture both long-range dependencies and multi-scale regulatory relationships within genomic sequences. The DPGM enhances feature selection by dynamically gating and refining input representations, thereby improving the model’s ability to focus on biologically relevant signals. Finally, the ResCNNChn employs a sequence of residual CNN blocks to extract high-level feature representations, further boosting predictive performance while maintaining computational efficiency. By combining these complementary components, MTMixG-Net provides a unified framework capable of modeling complex regulatory interactions in plant genomics, achieving both superior accuracy and scalability in gene expression prediction. Our key contributions are as follows:

- We present MTMixG-Net, a novel deep learning framework that integrates Transformer and Mamba architectures with a dual-path gating mechanism, specifically designed for plant gene expression prediction.
- We design the MTMixEnc module to jointly capture long-range dependencies and multi-scale genomic relationships, overcoming limitations of conventional CNN- or Transformer-based approaches.
- We introduce the dual-path gating mechanism (DPGM), which dynamically refines feature selection and enhances the model’s focus on biologically relevant regulatory signals.
- We implement the residual CNN chain (ResCNNChn) to extract high-level representations while maintaining computational efficiency, thereby boosting predictive performance.
- We validate MTMixG-Net on multiple plant genomic datasets and demonstrate that it consistently outperforms state-of-the-art baselines in both predictive accuracy and efficiency.

## 2 Materials and methods

### 2.1 Materials

To evaluate the effectiveness of MTMixG-Net, we conduct experiments on datasets from four diverse crop species.

Arabidopsis thaliana (A. tha). This dataset includes the reference genome spanning all 5 chromosomes, an annotation file with 27,655 genes, and corresponding transcription start sites (TSS) and transcription termination sites (TTS), as well as leaf tissue expression profiles.

Solanum lycopersicum (S. lyc). This dataset contains the reference genome with 10 chromosomes, annotations for 34,658 genes, and leaf tissue expression data.

Sorghum bicolor (S. bic). This dataset provides the reference genome with 12 chromosomes, annotations for 34,118 genes, and leaf tissue expression data, including TSS and TTS information.

Zea mays (Z. may). This dataset includes the reference genome with 10 chromosomes, annotations for 39,757 genes, and leaf tissue expression data.

The reference genomes and gene annotations for all species are obtained from the Ensembl Plants database^^1^^.

Data Processing. Genomic regions are extracted from 1 kb upstream to 0.5 kb downstream of each TSS and from 0.5 kb upstream to 1 kb downstream of each TTS, which serve as the model inputs. For transcriptomic data, short-read RNA-seq datasets are downloaded from the NCBI Sequence Read Archive (NCBI-SRA) (Wang et al., 2022) using fasterq-dump. Raw reads are trimmed using Sickle and aligned to the reference cDNA sequences with Kallisto. Gene expression quantification is performed using the tximport package in R, producing standardized counts expressed as transcripts per million (TPM), following the pipeline described in DeepPlantCRE (Wu et al., 2025).

Label Construction. For each species, genes are stratified into three expression categories based on the distribution of logMaxTPM values: low expression (logMaxTPM ≤ 25%, labeled as -1), medium expression (logMaxTPM between 25% and 75%, labeled as 0), and high expression (logMaxTPM ≥ 75%, labeled as 1).

### 2.2 Methods

**Figure 1:**
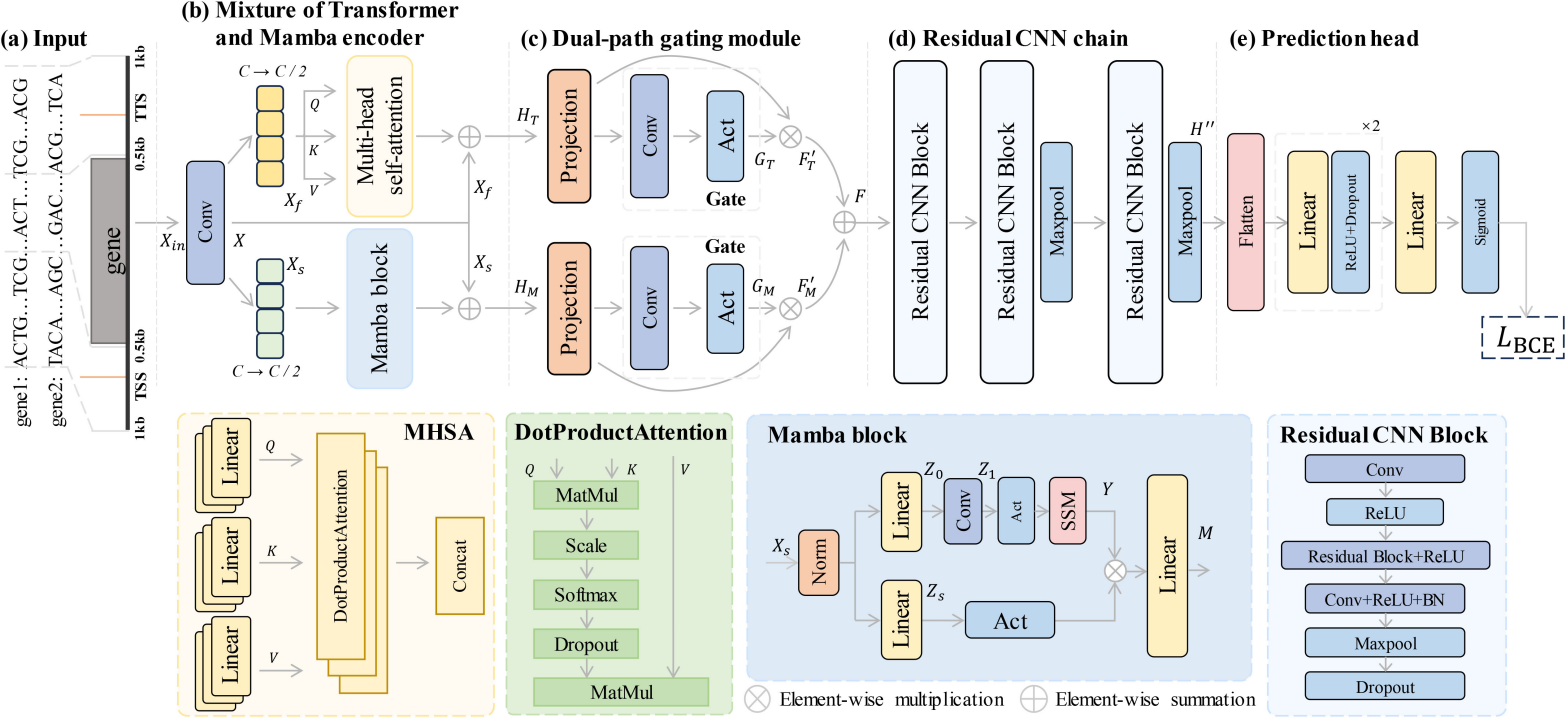
Framework architecture for gene expression prediction. **(a)** The input sequence, consisting of genomic data, is processed through a mixture of Transformer and Mamba encoders. **(b)** The encoder integrates multi-headed self-attention (MHSA) and Mamba blocks to capture both long-range dependencies and hierarchical patterns. **(c)** A dual-path gating mechanism selectively filters features to emphasize relevant information. **(d)** The residual CNN chain processes the features through multiple residual CNN blocks to extract high-level representations. **(e)** The prediction head generates the final gene expression predictions using fully connected layers and a binary cross-entropy loss function.

The overall architecture of MTMixG-Net is illustrated in **Figure 1**. The framework is composed of three key modules: the mixture of Transformer and Mamba encoder (MTMixEnc), the dual-path gating mechanism (DPGM), and the residual CNN chain (ResCNNChn). Given a genomic sequence as input, the MTMixEnc module first integrates the self-attention mechanism of Transformers with the hierarchical state-space structure of Mamba, enabling the model to capture both long-range dependencies and multi-scale regulatory relationships. The resulting representations are then refined by the DPGM, which applies a dynamic gating mechanism to selectively emphasize biologically relevant features. Finally, the ResCNNChn module employs a sequence of residual CNN blocks to extract high-level representations from the refined signals, leading to accurate predictions of gene expression.

### 2.3 Gene encoding

For each gene, the input sequence is constructed by extracting regions flanking both the TSS and the TTS. Specifically, we include 1 kb upstream to 0.5 kb downstream of the TSS, and 0.5 kb upstream to 1 kb downstream of the TTS. Each nucleotide (A, T, C, G) is encoded as a one-hot vector:


$$x_{i}\in {\left\{0,1\right\}}^{4},\;i=1,\;2,\ldots ,L$$

where *L* denotes the length of the input sequence. Accordingly, the entire sequence is represented as a matrix $X\in {\mathbb{R}}^{L\times 4}$.

### 2.4 Mixture of transformer and Mamba encoder

Modeling plant gene expression from genomic sequences requires capturing both long-range dependencies (e.g., distal enhancers influencing gene activity) and hierarchical integrative features (e.g., sequence motifs and cis-regulatory elements). Conventional CNNs are effective at detecting local patterns but fail to capture global interactions adequately. In contrast, Transformer architectures leverage self-attention to model long-range dependencies, yet they suffer from quadratic computational complexity and are prone to overfitting in data-limited genomic contexts. The recently proposed Mamba framework (Gu and Dao, 2023; Dao and Gu, 2024) addresses some of these limitations by integrating state-space models (SSMs) with convolutional layers, enabling efficient hierarchical modeling and scalable processing of long genomic sequences. However, Mamba alone may underperform in capturing complex global dependencies compared to attention-based mechanisms. To combine the strengths of both, we propose the mixture of Transformer and Mamba encoder (MTMixEnc), which integrates Transformer-based multi-head self-attention with Mamba blocks. This hybrid architecture enables the model to capture both fine-grained and multi-scale regulatory patterns, while maintaining computational efficiency and robustness in genomic applications.

Specifically, given an input sequence embedding $X_{in}\in {\mathbb{R}}^{L\times d}$, where *d* = 4 denotes the embedding dimension, the MTMixEnc encoder first applies a 1D convolutional layer with kernel size 1 to extract low-level features *X_conv_* ∈ ℝ*^L^*^×^*^C^*, where *C* = 64 is the channel size. The resulting feature map is then evenly split along the channel dimension into two parts, each with size ${\mathbb{R}}^{L\times C/2}$, which is subsequently forwarded to the parallel branches for further processing:

#### 2.4.1 Multi-head self-attention

The MHSA branch captures global dependencies across genomic positions. The first half of the input, $X_{f}\in {\mathbb{R}}^{L\times C/2}$, is projected into query (*Q*), key (*K*), and value (*V)* representations:


$$Q=X_{f}W_{Q},\;K=X_{f}W_{K},\;V=X_{f}W_{V},$$

where $W_{Q},W_{K},W_{V}\in {\mathbb{R}}^{C/2\times d_{k}}$ are trainable projection matrices and *d_k_* is the head dimension. The scaled dot-product attention is defined as:


$$\text{Attention}(Q,K,V)=\text{softmax}(\frac{QK^{T}}{\sqrt{d_{k}}})V.$$

For *h* attention heads, the multi-head formulation is:


$$\text{MHSA}(X)=\text{Concat}({\text{head}}_{1},\ldots ,{\text{head}}_{h})W_{O},$$

with ${\text{head}}_{i}=\text{Attention}(Q_{i},K_{i},V_{i})$ and $W_{O}\in {\mathbb{R}}^{h\times d_{k}\times C/2}$. This mechanism allows the model to weigh nucleotides across positions, thereby capturing long-range regulatory dependency between distant regulatory elements.

#### 2.4.2 Hierarchical state-space modeling

The second half of the feature, $X_{s}\in {\mathbb{R}}^{L\times C/2}$, is fed into the Mamba branch. Before feature extraction, the feature *X_s_* is normalized and linearly projected:


$$X_{s}^{'}=\text{Norm}(X_{s}),\;Z_{0}=X_{s}^{'}W_{1}+b_{1},$$

where $W_{1}\in {\mathbb{R}}^{C/2\times d_{h}},b_{1}\in {\mathbb{R}}^{d_{h}}$, and $d_{h}$ is the hidden dimension. A 1D convolutional layer is applied to capture local dependencies such as short motifs near transcription start or termination sites:


$$Z_{1}=\sigma (\text{Conv}1\text{D}(Z_{0})),$$

where *σ* is a non-linear activation function (e.g., ReLU).

To capture global and hierarchical sequence dynamics, we apply an SSM layer. Formally, an SSM can be written as:


$$h_{t}=Ah_{t-1}+Bx_{t},y_{t}=Ch_{t}+Dx_{t},$$

where $h_{t}\in {\mathbb{R}}^{d_{s}}$ is the hidden state at time t, $x_{t}\in {\mathbb{R}}^{d_{h}}$ is the input, $y_{t}\in {\mathbb{R}}^{d_{h}}$ is the output, and *A, B, C, D* are learned parameters. In practice, this recurrence can be efficiently expressed as a state-space convolution:


$$Y=(B*X_{s})+(A*H),$$

where ∗ denotes convolution.

In parallel, the normalized feature $X_{s}^{\prime }$ is linearly projected and passed through a non-linear activation:


$$Z_{s}=\sigma (X_{s}^{\prime }W_{s}+b_{s}),$$

where $W_{s}\in {\mathbb{R}}^{C/2\times d_{h}},b_{s}\in {\mathbb{R}}^{d_{h}}$.

Finally, the outputs from the SSM and the parallel branch are combined via element-wise multiplication, followed by a linear mapping, to obtain the final Mamba representation:


$$M=(Y⊗Z_{s})W_{2}+b_{2},$$

where ⊗ denotes element-wise multiplication, *σ*(·) is a non-linear activation, and $M\in {\mathbb{R}}^{L\times C/2}$ is the final output of the Mamba branch. This design enables hierarchical representation learning of motifs and regulatory patterns, complementing the global attention captured by the Transformer branch.

#### 2.4.3 The final outputs of MHSA and Mamba

After processing the input sequence through both the Transformer and Mamba branches, their outputs are fused with the convolutional features to integrate local motif information. Specifically, the output of the MHSA branch is denoted as *H*_T_, and the output of the Mamba branch as *H*_M_:


$$H_{\text{T}}=X_{f}⊕\text{MHSA}(X),\;H_{\text{M}}=X_{s}⊕M,$$

where ⊕ denotes element-wise addition.

This fusion strategy enables the model to jointly leverage (i) global contextual information captured by the MHSA branch and (ii) hierarchical sequence features extracted by the Mamba block, while grounding both representations in the local motif patterns encoded by the convolutional layer. Such integration enhances the model’s ability to capture complex regulatory interactions in genomic sequences.

### 2.5 Dual-path gating mechanism

Although the MTMixEnc captures long-range dependencies and hierarchical structures, its outputs may still contain redundant or noisy features that are not directly relevant to gene expression prediction. To address this, we introduce the DPGM, which selectively emphasizes biologically informative signals while suppressing irrelevant activations. Unlike single-path gating, DPGM employs two parallel gating streams, allowing the model to learn complementary feature filters and adaptively balance motif-level and global contextual information.

Formally, given the outputs of MTMixEnc, $H_{\text{T}},H_{\text{M}}\in {\mathbb{R}}^{L\times C/2}$, the gating masks are computed as:


$$G_{\text{T}}=\sigma (\text{Conv}(\text{Project}(H_{\text{T}}))),\;G_{\text{M}}=\sigma (\text{Conv}(\text{Project}(H_{\text{M}}))),$$

where Project(·) denotes a linear projection for dimensionality reduction, Conv(·) is a 1D convolution, and *σ* is a sigmoid function producing gating values in [0,1].

The final gated outputs, *F*_T_ and *F*_M_, are obtained by element-wise multiplication of the original features with their respective gating masks:


$$F_{\text{T}}^{\prime }=\text{Project}(H_{\text{T}})⊗G_{\text{T}},\;F_{\text{M}}^{\prime }=\text{Project}(H_{\text{M}})⊗G_{\text{M}}.$$

This operation allows the model to retain important features while suppressing less relevant ones.

Finally, the outputs from the two paths are combined to form the final feature representation *F* that will be passed to the next module:


$$F=F_{\text{T}}^{\prime }⊕F_{\text{M}}^{\prime },$$

where the addition operation allows the model to integrate complementary information from both gating paths. By combining dual gating paths, DPGM enhances the model’s ability to retain biologically relevant signals and suppress redundant noise, thereby improving predictive robustness.

### 2.6 Residual CNN chain

Although the MTMixEnc captures long-range dependencies and hierarchical structures, the resulting feature space may still lack fine-grained local patterns critical for distinguishing subtle regulatory elements. Moreover, deep CNNs are prone to vanishing gradients and performance degradation as depth increases. To address these issues, we employ a ResCNNChn, where stacked convolutional layers are stabilized with residual connections (He et al., 2016). This design enables deeper networks, preserves gradient flow, and facilitates the extraction of high-level local regulatory representations.

Given the refined representation $F\in {\mathbb{R}}^{L\times C/2}$ from the DPGM, ResCNNChn processes it through a sequence of residual CNN blocks, optionally interleaved with max-pooling layers (**Figure 1d**):


$$H^{\prime \prime }={\text{ResBlock}}_{n}(\text{MaxPool}(\ldots {\text{ResBlock}}_{2}(\text{MaxPool}({\text{ResBlock}}_{1}(F))))),$$

where $H^{\prime \prime }\in {\mathbb{R}}^{L^{\prime }\times C^{\prime }}$ is the final output of the ResCNNChn, *n* is the number of residual blocks, and MaxPool(·) is an optional max-pooling layer that reduces the sequence length while retaining salient features.

Each residual CNN block ResBlock*_i_* consists of convolutional layers with non-linear activations, residual connections, batch normalization, and dropout:


$$\begin{array}{l} Z_{1}=\sigma (\text{Conv}1\text{D}(H_{in})),\\ Z_{2}=\sigma (\text{ConvlD}(Z_{1}))+H_{in},\\ Z_{3}=\text{BN}(\sigma (\text{Conv}1\text{D}(Z_{2}))),\\ Z_{4}=\text{MaxPool}(Z_{3}),\\ H_{out}=\text{Dropout}(Z_{4},p), \end{array}$$

where $H_{in}\in {\mathbb{R}}^{L_{in}\times C_{in}}$ is the input feature map for each ${\text{ResBlock}}_{i}$, $H_{out}\in {\mathbb{R}}^{L_{out}\times C_{out}}$ is the output feature map, *σ* is a non-linear activation function (e.g., ReLU), BN(·) is batch normalization, MaxPool(·) is max-pooling, and Dropout(·) applies dropout to prevent overfitting. It is noted that each residual CNN block outputs $H_{out}\in {\mathbb{R}}^{L_{out}\times C_{out}}$, ready to be passed to the next block in the chain.

Through this residual design, ResCNNChn preserves fine-grained local patterns, stabilizes deeper architectures, and enhances the overall representation power for modeling complex regulatory signals.

### 2.7 Prediction head

The final feature maps are flattened and passed through a series of fully connected layers with ReLU activations and dropout regularization to produce the predictive logits *Z_logits_* (**Figure 1e**). The output layer then applies a sigmoid activation to generate class probabilities for gene expression levels:


$$\hat{y}=\text{Sigmoid}(Z_{logits}),$$

where $\hat{y}\in {\mathbb{R}}^{Cls}$ denotes the predicted probability distribution over *Cls* discrete gene expression classes (e.g., low, medium, high).

### 2.8 Loss function

We employ binary cross-entropy (BCE) loss to supervise the classification of gene expression levels (low, medium, high). In our formulation, each class is represented as a one-hot vector, and BCE is applied independently to each class dimension:


$$L_{\text{BCE}}=-\frac{1}{N}\sum_{i=1}^{N}\sum_{c=1}^{Cls}[y_{i,c}\text{ log}({\hat{y}}_{i,c})+\;(1-y_{i,c})\;\text{log}(1-{\hat{y}}_{i,c})],$$

where *N* is the number of samples, *Cls* = 3 is the number of classes, *y_i,c_* ∈ 0, 1 is the ground-truth indicator for class c, and ${\hat{y}}_{i,c}$ is the predicted probability. This formulation encourages the model to assign high probability to the correct class while penalizing incorrect predictions, thereby improving classification accuracy.

## 3 Experiments and results

### 3.1 Experimental settings

#### 3.1.1 Evaluation metrics

To evaluate the predictive performance of our models, we employ three widely used metrics: Accuracy, AUC-ROC, and F1-score. Accuracy measures the proportion of correctly predicted samples out of the total, providing an overall assessment of model performance. We use the AUC-ROC curve, where the horizontal axis represents the false positive rate and the vertical axis represents the true positive rate. The AUC indicates the model’s ability to distinguish between the positive and negative classes. The F1-score is the harmonic mean of precision and recall, balancing both metrics to evaluate the model’s ability to correctly classify gene expression levels while minimizing false positives and negatives. These metrics are computed based on the counts of true positives (TP), true negatives (TN), false positives (FP), and false negatives (FN): TP refers to correctly identified positive samples, TN to correctly identified negative samples, FP to negative samples incorrectly classified as positive, and FN to positive samples incorrectly classified as negative. The formulas for these metrics are as follows:


$$\begin{array}{l} \text{Accuracy}=\frac{\text{TP}+\text{TN}}{\text{TP}+\text{TN}+\text{FP}+\text{FN}},\\ \text{Pre}=\frac{\text{TP}}{\text{TP}+\text{FP}},\\ \text{Sen}=\frac{\text{TP}}{\text{TP}+\text{FN}},\\ \text{F}1=2\times \frac{\text{Pre}\times \text{Sen}}{\text{Pre}+\text{Sen}}. \end{array}$$

#### 3.1.2 Implementation

All experiments are conducted on an NVIDIA 3090 GPU. For training our model, MTMixG-Net, we use the Adam optimizer with an initial learning rate of 10^−4^. The training is limited to a maximum of 100 epochs, with each epoch consisting of forward propagation, back-propagation, and parameter updates on the training data. To mitigate overfitting, early stopping is employed, halting training if the validation loss does not decrease for 10 consecutive epochs. Additionally, the learning rate is dynamically adjusted: it is reduced to 10% of its current value if no improvement in validation loss is observed for 5 consecutive epochs. This dynamic adjustment helps the model converge more effectively in the later stages of training.

To ensure robustness, we adopt *k*-fold cross-validation, with *k* set equal to the number of chromosomes for each dataset. All models are trained and evaluated under the same conditions, using identical training, validation, and test splits to ensure a fair comparison. Each experiment is repeated 5 times with different random seeds to account for variability in training, and the average results along with standard deviations are reported.

### 3.2 Comparison with state-of-the-art models

To evaluate the performance of our proposed MTMixG-Net, we compare it with three representative deep learning models: DeepCRE (Peleke et al., 2024), PhytoExpr (Li et al., 2024), and DeepPlantCRE (Wu et al., 2025) across four plant species datasets. The DeepCRE employs CNNs to analyze genomic sequences, predict gene expression levels, and identify cis-regulatory elements. PhytoExpr extends CNN-based architectures by integrating Transformer layers, aiming to capture long-range dependencies in genomic data and improve prediction accuracy for mRNA abundance. DeepPlantCRE further enhances the modeling of cis-regulatory elements by combining CNNs with deeper sequence encoders, achieving competitive results on multiple plant datasets. Since DeepPlantCRE provides publicly available code, we reimplement it for our experiments; for the other two methods, which lack open-source implementations, we adopt the performance reported in (Wu et al., 2025).

The results are summarized in **Table 1**. For A. tha, DeepPlantCRE achieves the second-best results across all three metrics (Accuracy: 86.1%, AUC-ROC: 93.0%, F1-score: 85.8%). MTMixG-Net consistently outperforms all baselines, with the best Accuracy (87.4%), AUC-ROC (93.7%), and F1-score (87.1%). In the S. lyc dataset, DeepPlantCRE again deliveres strong results (Accuracy: 84.3%, AUC-ROC: 90.0%, F1-score: 77.9%), but MTMixG-Net surpasses it with the highest Accuracy (85.4%), AUC-ROC (91.0%), and F1-score (78.5%). For S. bic, DeepPlantCRE showes second-best performance (Accuracy: 80.7%, AUC-ROC: 86.2%, F1-score: 76.1%). MTMixG-Net clearly outperformes the baselines with Accuracy of 81.8%, AUC-ROC of 87.1%, and F1-score of 77.4%. On the Z. may dataset, DeepPlantCRE again rankes second (Accuracy: 83.0%, AUC-ROC: 90.0%, F1-score: 81.2%), while MTMixG-Net achieves the best results with Accuracy (84.1%), AUC-ROC (90.1%), and F1-score (82.1%).

**Table 1:** Performance comparison of all the models on four datasets. .

Datasets	Metrics	DeepCRE	PhytoExpr	DeepPlantCRE	MTMixG-Net
A. tha	Accuracy	85.4 ± 2.0	78.3 ± 1.5	86.1 ± 3.0	**87.4 ± 2.1**
	AUC-ROC	92.5 ± 1.8	86.3 ± 0.9	93.0 ± 1.7	**93.7 ± 1.1**
	F1-score	85.0 ± 1.9	76.6 ± 2.4	85.8 ± 2.8	**87.1 ± 2.3**
S. lyc	Accuracy	82.6 ± 2.7	79.2 ± 3.0	84.3 ± 2.6	**85.4 ± 2.7**
	AUC-ROC	87.7 ± 2.4	85.2 ± 3.1	90.0 ± 2.3	**91.0 ± 2.1**
	F1-score	74.7 ± 4.5	71.3 ± 5.5	77.9 ± 3.9	**78.5 ± 3.7**
S. bic	Accuracy	79.0 ± 1.9	75.7 ± 2.4	80.7 ± 1.6	**81.8 ± 2.2**
	AUC-ROC	84.4 ± 5.3	81.9 ± 2.3	86.2 ± 4.8	**87.1 ± 5.3**
	F1-score	73.5 ± 6.9	71.5 ± 5.2	76.1 ± 6.4	**77.4 ± 6.4**
Z. may	Accuracy	80.1 ± 3.9	77.9 ± 4.1	83.0 ± 4.5	**84.1 ± 2.7**
	AUC-ROC	86.7 ± 5.1	85.2 ± 4.0	90.0 ± 4.1	**90.1 ± 3.6**
	F1-score	78.0 ± 5.8	76.2 ± 5.1	81.2 ± 6.1	**82.1 ± 4.0**

The best values are highlighted in bold. The second-best values are marked in red.

These results demonstrate that MTMixG-Net consistently outperforms existing state-of-the-art models, effectively capturing the complex regulatory patterns underlying gene expression in plants. Moreover, the consistent improvements across species with large and complex genomes highlight the robustness and generalizability of MTMixG-Net in handling redundant regulatory sequences.

### 3.3 Ablation study

To assess the contributions of each component in MTMixG-Net, we conduct an ablation study by systematically adding key modules and evaluating the impact on performance. We consider three configurations: (1) ResCNNChn only (*M*_1_): A baseline using only the residual CNN chain without MTMixEnc or DPGM. This configuration evaluates the effectiveness of local motif extraction via CNN blocks. (2) ResCNNChn + MTMixEnc(*M*_1_+*M*_2_): Incorporates the MTMixEnc module together with ResCNNChn, excluding DPGM. This setting tests the impact of combining self-attention and state-space modeling for global and hierarchical feature learning. For the MTMixEnc, we experiment with two variants: (*M*_21_) using only the Mamba branch, and (*M*_22_) using only the Transformer branch. (3) Full MTMixG-Net (*M*_1_+*M*_2_+*M*_3_): The complete model including ResCNNChn (*M*_1_), MTMixEnc (*M*_2_), and DPGM (*M*_3_).

**Figure 2:**
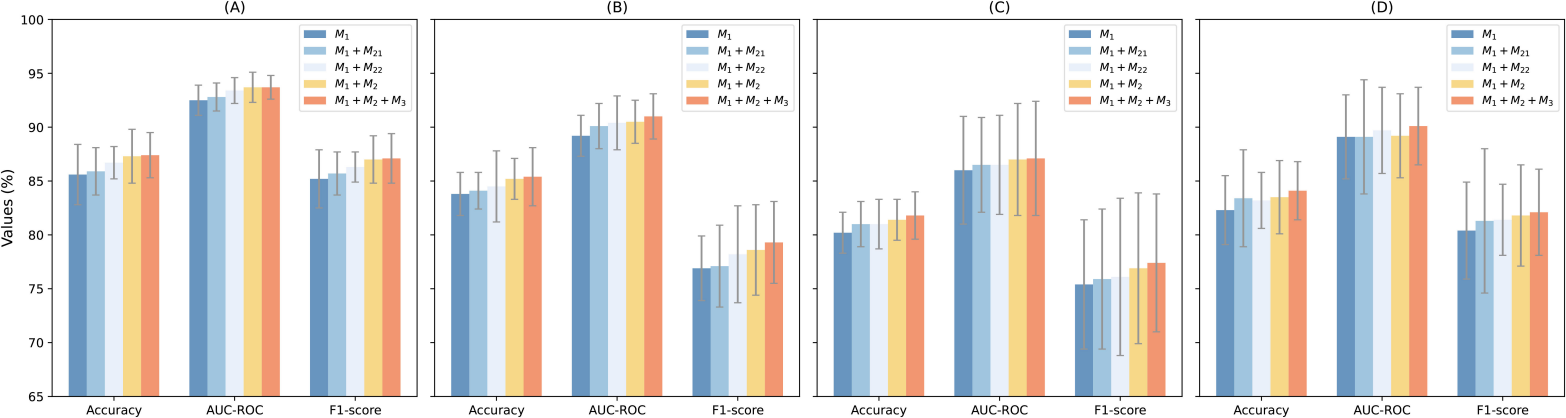
Performance visulizion of the ablation studies on the **(A)** arabidopsis thaliana, **(B)** solanum lycopersicum, **(C)** sorghum bicolor, and **(D)** zea mays datasets.

The results (**Table 2**; **Figure 2**) show that even ResCNNChn alone achieves competitive performance, with accuracies between 80.2% (S. bic) and 85.6% (A. tha), demonstrating the effectiveness of residual CNN blocks in extracting local sequence patterns. However, the lack of global modeling limits accuracy in species with complex regulatory architectures.

**Table 2:** Ablation experiments on the four datasets.

Module	A. tha		S. lyc		S. bic		Z. may	
	Accuracy	AUC-ROC	Accuracy	AUC-ROC	Accuracy	AUC-ROC	Accuracy	AUC-ROC
*M*_1_	85.6 ± 2.8	92.5 ± 1.4	83.8 ± 2.0	89.2 ± 1.9	80.2 ± 1.9	86.0 ± 5.0	82.3 ± 3.2	89.1 ± 3.9
*M*_1_+*M*_21_	85.9 ± 2.2	92.8 ± 1.3	84.1 ± 1.7	90.1 ± 2.1	81.0 ± 2.1	86.5 ± 4.4	83.4 ± 4.5	89.1 ± 5.3
*M*_1_+*M*_22_	86.7 ± 1.5	93.4 ± 1.2	84.5 ± 3.3	90.4 ± 2.5	81.0 ± 2.3	86.5 ± 4.6	83.2 ± 2.6	89.7 ± 4.0
*M*_1_+*M*_2_+	87.3 ± 2.5	93.7 ± 1.4	85.2 ± 1.9	90.5 ± 2.0	81.4 ± 1.9	87.0 ± 5.2	83.5 ± 3.4	89.2 ± 3.9
*M*_1_+*M*_2_+*M*_3_	87.4 ± 2.1	93.7 ± 1.1	85.4 ± 2.7	91.0 ± 2.1	81.8 ± 2.2	87.1 ± 5.3	84.1 ± 2.7	90.1 ± 3.6

Incorporating the MTMixEnc improves performance across nearly all datasets. For example, in A. tha, accuracy improves from 85.6% to 87.3%, and in S. bic, accuracy increases from 80.2% to 81.4%. The AUC-ROC also rises slightly (e.g., 86.0% → 87.0% in S. bic). When combined individually with the *M*_1_, both *M*_1_+*M*_21_ and *M*_1_+*M*_22_ outperform the CNN-only baseline across all datasets, confirming that integrating either sequential modeling mechanism enhances the representation of genomic dependencies. Notably, *M*_1_+*M*_21_ (Mamba-based) achieves slightly higher Accuracy and AUC-ROC values than *M*_1_+*M*_22_ (Transformer-based) in most species, indicating that self-attention remains highly effective for capturing long-range dependencies. However, the performance gap between the two configurations is marginal (≤0.5%), suggesting that Mamba provides a computationally efficient alternative capable of modeling hierarchical temporal features with comparable accuracy. These gains highlight that combining Transformer’s ability to capture long-range dependencies with Mamba’s hierarchical modeling enhances representation learning beyond local CNN features.

Incorporating DPGM further refines the learned representations. By suppressing redundant features and highlighting biologically relevant signals, DPGM provides consistent improvements over A+C. For example, in S. lyc, accuracy rises from 85.2% to 85.4%, while AUC-ROC increases from 90.5% to 91.0%. Similar enhancements are observed across all datasets.

The ablation results confirm that each module contributes meaningfully to overall performance. The residual CNN chain captures strong local patterns, the MTMixEnc enhances global and hierarchical feature learning, and the DPGM further filters and amplifies informative signals. The full MTMixG-Net consistently achieves the best performance across datasets, validating the necessity of integrating all three components for accurate and generalizable plant gene expression prediction.

### 3.4 Hyperparameter selection

**Figure 3:**
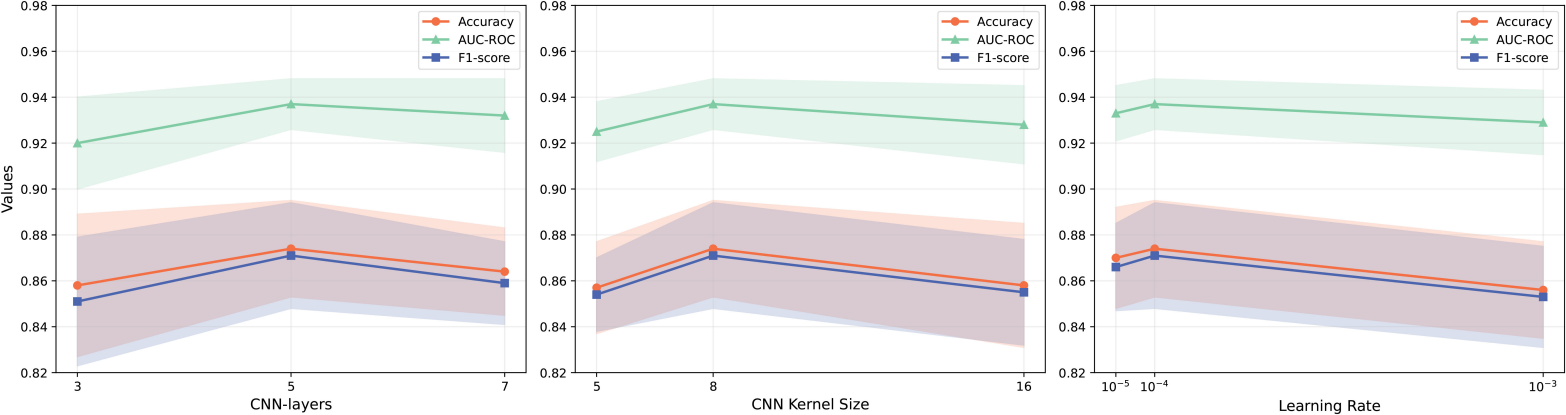
Hyperparameter sensitivity analysis of MTMixG-Net.

Selecting appropriate hyperparameters is critical to ensure both the stability and effectiveness of MTMixGNet. To systematically evaluate their influence, we vary the number of CNN layers, kernel sizes, and learning rates, and assess performance using accuracy, AUC-ROC, and F1-score (**Figure 3**).

We experiment with 3, 5, and 7 CNN layers. Performance peaks at 5 layers, where accuracy and F1-score both achieve their highest values. Using fewer layers (3) lead to underfitting, as the model lacks sufficient representational depth. Increasing to 7 layers does not improve performance and slightly reduced AUCROC, suggesting potential overfitting and gradient instability. Therefore, 5 CNN layers strike a balance between expressive power and generalization.

Kernel sizes of 5, 8, and 16 are compared. A kernel size of 8 provides the best trade-off, with the highest AUC-ROC and F1-score. Smaller kernels (size 5) failes to capture wider motifs effectively, while overly large kernels (size 16) dilutes local features and reduces precision. This result indicates that medium-sized kernels are most effective for modeling cis-regulatory elements in plant genomic data.

We test learning rates of 10^−5^, 10^−4^, and 10^−3^. A learning rate of 10^−4^ produces the most stable and optimal results, with the highest accuracy and AUC-ROC. At 10^−5^, the model converges slowly, leading to suboptimal results within the given training epochs. In contrast, 10^−3^ causes performance degradation, likely due to unstable weight updates. Thus, 10^−4^ is selected as the optimal learning rate for all experiments.

The different hyperparameter settings demonstrates that MTMixG-Net is sensitive to architectural and optimization choices. Specifically, moderate network depth (5 layers), medium kernel sizes (8), and balanced learning rates (10^−4^) yield the most robust and generalizable performance across datasets. These settings ensure the model captures both local motif structures and long-range dependencies without overfitting, validating the importance of careful hyperparameter tuning in genomic prediction tasks.

### 3.5 Cross-species generalization

**Figure 4:**
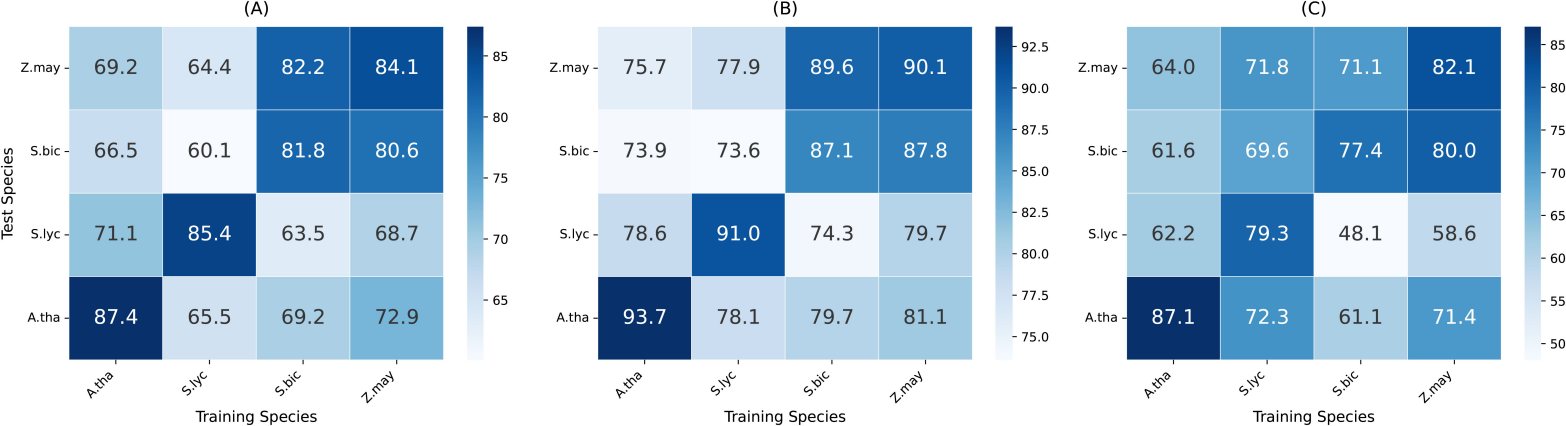
Cross-species generalization performance of MTMixG-Net on the four datasets. **(A)** Accuracy, **(B)** AUC-ROC, **(C)** F1-score.

One of the key challenges in plant genomics is generalizing across species with diverse genomic structures and regulatory mechanisms. To assess the cross-species generalization capability of MTMixG-Net, we conduct experiments where the model is trained on one species and tested on others. The results are summarized in the heatmaps shown in **Figure 4**.

MTMixG-Net generally performs well across species, particularly when trained on zea mays and tested on other species. Notably, the zea mays dataset, with 39,757 gene records, is significantly larger than those of the other species, presenting greater complexity. This larger dataset may cause the model to overfit the more complex gene set, which could explain the observed challenges when training on zea mays and testing on other species. In contrast, smaller datasets from species like solanum lycopersicum or arabidopsis thaliana may provide fewer but more transferable regulatory signals, enabling easier generalization.

However, MTMixG-Net performs the worst when trained or tested on the sorghum bicolor dataset, particularly in terms of accuracy, AUC-ROC, and F1-score. This anomaly suggests challenges in handling sorghum bicolor genomic data. We explain this phenomenon from two perspectives. First, sorghum bicolor has a relatively small dataset size (only 17,988 gene records), which may limit the model’s ability to learn robust and generalizable features. Second, sorghum bicolor’s genomic architecture may contain unique regulatory elements or patterns that are not well-represented in the training data from other species, leading to poor performance when the model is applied to this species.

In summary, these results demonstrate that MTMixG-Net exhibits strong cross-species generalization. The model’s ability to maintain high accuracy, AUC-ROC, and F1-score in cross-species settings indicates its capacity to capture conserved regulatory patterns rather than overfitting to species-specific signals. This adaptability makes MTMixG-Net a valuable tool for plant genomics, especially for crops with limited annotated datasets.

## 4 Conclusion

In this study, we propose MTMixG-Net, a novel deep learning framework for plant gene expression prediction that jointly captures multi-scale and global regulatory patterns from genomic sequences. By integrating a mixture of Transformer and Mamba encoder (MTMixEnc), a dual-path gating mechanism (DPGM), and a residual CNN chain (ResCNNChn), the model effectively overcomes the limitations of prior approaches that consider local motifs and long-range dependencies in isolation. Extensive experiments on four plant species datasets demonstrate that MTMixG-Net consistently outperforms state-of-the-art baselines. Ablation studies further validate the contribution of each module, while cross-species experiments highlight the model’s robustness and versatility, underscoring its ability to generalize across genomes with diverse architectures. Overall, MTMixG-Net represents a significant advancement in computational genomics, offering a powerful tool for understanding and predicting gene expression in plants. Its strong performance and generalizability make it a promising approach for future applications in plant biology and crop improvement. In future work, MTMixG-Net could be extended to integrate transcriptomic, epigenetic, and environmental data, facilitating its application in real-world crop improvement and functional genomics. Translating such models into practical breeding tools, however, will require overcoming challenges related to data heterogeneity, interpretability, and the need for large-scale, high-quality annotations across diverse plant species.

## Data Availability

The datasets presented in this study can be found in online repositories. The names of the repository/repositories and accession number(s) can be found in the article/supplementary material.

## Footnotes

1 plants.ensembl.org
